# Differential Signature of Gut Microbiota in Female Patients with Condyloma Acuminatum and Its Association with Persistent Human Papillomavirus Infection

**DOI:** 10.3390/jcm15187265

**Published:** 2026-09-18

**Authors:** Yinhua Wu, Qiujun Zhou, Chen Wang, Xiaoyan Liu

**Affiliations:** Department of Dermatology, The 1st Affiliated Hospital of Zhejiang University School of Medicine, Hangzhou 310003, China

**Keywords:** condyloma acuminatum, gut microbiota, HPV persistence, 16S rRNA sequencing, *Bacteroides vulgatus*, *Bacteroides stercoris*, microbial biomarkers

## Abstract

**Background:** Human papillomavirus (HPV) persistence is a critical driver of malignant transformation, yet the potential association between gut microbiota composition and HPV clearance remains insufficiently explored. **Methods:** This study investigated the association between gut microbial features and HPV infection outcomes in patients with condyloma acuminatum (CA). Baseline fecal samples from 44 CA patients and 20 healthy controls underwent 16S rRNA gene sequencing, followed by a two-year clinical follow-up to stratify patients into HPV turn-negative (HPV_TN) and persistent-positive (HPV_PP) groups. Bioinformatic analyses, including alpha/beta diversity metrics, linear discriminant analysis Effect Size (LEfSe), Phylogenetic Investigation of Communities by Reconstruction of Unobserved States (PICRUSt2) functional prediction, and receiver operating characteristic (ROC) curve evaluation, were employed. **Results:** CA patients exhibited significantly increased microbial richness and distinct beta diversity compared to controls. Notably, LEfSe analysis identified *Bacteroides stercoris* as a significant biomarker enriched in the HPV_PP group, while *Bacteroides vulgatus* showed nominally higher abundance in the HPV_PP group. ROC analysis yielded area under the curves (AUCs) of 0.733 for *B. stercoris* and 0.729 for *B. vulgatus*, with a combined two-species logistic regression model achieving an AUC of 0.743. Furthermore, Mantel’s test and Spearman correlation analysis linked specific microbial shifts to clinical parameters including HPV persistence status. **Conclusions:** This exploratory study identifies associations between gut microbiota composition and HPV persistence in CA patients. *B. stercoris* represents the most robust candidate microbial signature, while *B. vulgatus* provides supplementary evidence. These findings generate hypotheses for future prospective and mechanistic studies investigating whether gut microbiota modulation could influence HPV clearance. The enrichment of *B. stercoris* and *B. vulgatus* may represent candidate microbial signatures requiring independent external validation.

## 1. Introduction

Condyloma acuminatum (CA), caused by human papillomavirus (HPV), represents one of the most common sexually transmitted conditions worldwide, posing considerable clinical challenges owing to its tendency for recurrence and the risk of prolonged viral carriage [1]. Although current therapeutic strategies predominantly target lesion ablation, achieving HPV elimination remains the cornerstone for preventing relapse and malignant progression. Reported recurrence rates after CA treatment range from 10% to 70%, and a substantial proportion of patients exhibit persistent HPV detection at follow-up, with persistence rates varying by anatomical site, HPV genotype, and follow-up duration [1,2]. The transition from acute infection to viral persistence is governed by complex host–microbe interactions, yet the precise determinants of this process remain poorly defined [2]. Mounting evidence indicates that mucosal and systemic microbial communities play pivotal roles in shaping antiviral immune defenses [3,4].

The intestinal microbiome has emerged as a central orchestrator of host immunity, capable of modulating systemic inflammatory cascades and antiviral defense programs via the gut–mucosal axis [5,6,7]. In particular, microbial-derived short-chain fatty acids (SCFAs) have been shown to calibrate both innate and adaptive antiviral responses [7,8]. In the context of HPV-associated diseases, dysbiosis within the vaginal microbiome—marked by depletion of protective Lactobacillus and expansion of anaerobic organisms—has been linked to viral persistence and neoplastic progression [9,10]. Specifically, studies have shown that a dysbiotic state characterized by loss of protective *Lactobacillus* species and overgrowth of anaerobic bacteria is associated with HPV persistence [11]. Additionally, animal models have revealed that gut microbial composition can influence the efficacy of HPV therapeutic vaccines through modulation of CD8+ T cell activity and immunosuppressive cell populations [12]. The recently proposed gut–vaginal axis further provides a biologically plausible framework for how intestinal microbes may remotely influence HPV-related outcomes [13], and intestinal dysbiosis has been implicated in a spectrum of gynecological conditions [14,15]. Collectively, these observations position the gut microbiome as a potentially modifiable determinant of HPV pathogenesis, although direct evidence in CA patients remains limited.

Despite these advances, a significant knowledge gap persists. Prior investigations have predominantly examined the vaginal or cervical microbiome, leaving the contribution of intestinal microbiota to CA pathogenesis—and specifically its relationship with post-treatment HPV persistence—largely uncharted. While studies in men who have sex with men have identified gut microbial signatures linked to persistent anal HPV infection [16], and recent work has begun to characterize microbiota disturbances in CA patients [17], no study has systematically profiled the gut microbiota in a general female CA cohort and correlated these profiles with clinical endpoints such as viral clearance or persistence. Addressing this gap could unveil candidate biomarkers for predicting therapeutic failure and generate hypotheses for novel intervention targets.

The specific aim of this exploratory study was to characterize the gut microbiota of patients with CA in comparison to healthy controls and to identify specific microbial taxa and predicted functional pathways associated with persistent HPV infection versus viral clearance following treatment. By delineating these microbial signatures, we sought to establish a foundational understanding of the potential gut–vaginal axis in CA and to explore the potential of gut microbiota-based candidate biomarkers for personalized risk stratification.

## 2. Materials and Methods

### 2.1. Study Design, Participants, and Sample Collection

The experimental design, participant selection criteria, clinical evaluations, HPV typing procedures, therapeutic interventions, and follow-up protocols were conducted as described comprehensively in our earlier publication [9]. Ethical approval was obtained from the ethics committee of the First Affiliated Hospital, Zhejiang University School of Medicine (approval no. 2020-057), with written informed consent provided by every participant prior to enrollment. For the current analysis, fecal specimens were procured from 44 of the 63 originally enrolled CA patients and 20 age-, body mass index (BMI)-, and marital status-matched healthy individuals. Samples were immediately placed in DNA stabilization reagent (TinyGene Bio-Tech Co., Ltd., Shanghai, China) and transferred to −80 °C storage within 15 min. Cervical HPV genotyping was performed at baseline and at 6-month intervals during the 2-year follow-up using the HPV GenoArray Test Kit (Hybribio, Chaozhou, China), which detects 25 HPV genotypes including 18 high-risk types. Participants were categorized into the HPV turn-negative (HPV_TN) group if HPV testing became negative within two years and remained negative for at least two consecutive follow-up visits or into the HPV persistent-positive (HPV_PP) group if at least one of the HPV types remained positive throughout the 2-year follow-up period.

### 2.2. 16S rRNA Sequencing and Bioinformatic Analysis

Genomic DNA isolation, amplification of the V3-V4 hypervariable segment using primer pair 341F/806R, sequencing library construction, and paired-end sequencing on the Illumina NovaSeq 6000 PE250 platform were all performed by TinyGene Bio-Tech Co., following the identical laboratory protocols established in our preceding study [9]. All raw sequencing reads have been deposited in the EMBL Sequence Read Archive under accession number PRJNA1033799.

The complete downstream analytical pipeline replicated that employed in our prior investigation [9]. In brief, high-quality reads were assigned to operational taxonomic units (OTUs) at a 97% identity threshold through the UPARSE algorithm (v8.1.1756) [18], with taxonomic classification performed using the SILVA 138.1 database [19] implemented in mothur (v1.39.5) [20]. Community richness was evaluated via Chao1 and ACE estimators and overall alpha diversity via Shannon, Simpson, and PD-whole tree indices. Beta diversity was assessed via principal component analysis (PCA) based on OTUs, principal coordinate analysis (PCoA) based on weighted UniFrac metrics, and non-metric multidimensional scaling (NMDS) based on Bray–Curtis dissimilarities, which were used for ordination visualization, and statistical significance of group separation in these ordinations was assessed via ANOSIM. Differentially represented taxa were pinpointed using linear discriminant analysis Effect Size (LEfSe) analysis [21], and functional metagenomic content was predicted with Phylogenetic Investigation of Communities by Reconstruction of Unobserved States (PICRUSt2) [22]. Associations between microbial profiles and clinical variables were assessed through Mantel’s test and Spearman’s rank correlation. Receiver operating characteristic (ROC) curve analysis [23] was conducted to appraise the exploratory diagnostic utility of candidate microbial indicators. For multi-species predictive modeling, logistic regression was applied using relative abundances of key species as input variables, with the area under the ROC curve (AUC) and 95% confidence intervals (CI) determined via 1000 bootstrap iterations. Given the modest sample size and the use of the same cohort for feature selection and model evaluation, the reported AUC values should be considered exploratory and may be subject to overfitting. Spearman rank correlation matrices among the top 20 species were calculated to capture co-occurrence patterns. All statistical analyses were executed in R (v3.6.3), with *p* < 0.05 as the significance cutoff. To address the issue of multiple hypothesis testing, Benjamini–Hochberg false discovery rate (FDR) correction was applied to the following analyses: (1) LEfSe biomarker identification and PICRUSt2 functional prediction, where adjusted *p*-values (*q*-values) were used in conjunction with the linear discriminant analysis (LDA) score threshold; (2) pairwise differential abundance testing of taxa at genus and species levels between study groups. For alpha diversity comparisons (Chao1, ACE, Shannon, Simpson, PD-whole tree), a modest number of pre-specified indices were compared, and raw *p*-values are reported with the caveat that these should be interpreted in light of multiple comparisons. ANOSIM tests for beta diversity ordinations are based on permutation procedures and were not additionally FDR-corrected given the small number of ordination methods tested. ROC curve analysis and logistic regression modeling were treated as exploratory given the limited sample size and lack of an independent validation cohort; accordingly, uncorrected *p*-values and AUC values are presented, and these results should be regarded as hypothesis-generating rather than confirmatory.

## 3. Results

### 3.1. CA Patients Exhibit Increased Microbial Richness and Distinct Gut Microbial Community Structure Compared with Healthy Controls

Detailed demographic and clinical characteristics of the 44 recruited CA patients are shown in Supplementary Table S1. No significant differences were found in age, BMI, or marital status between the CA and control (CON) groups.

**Figure 1:**
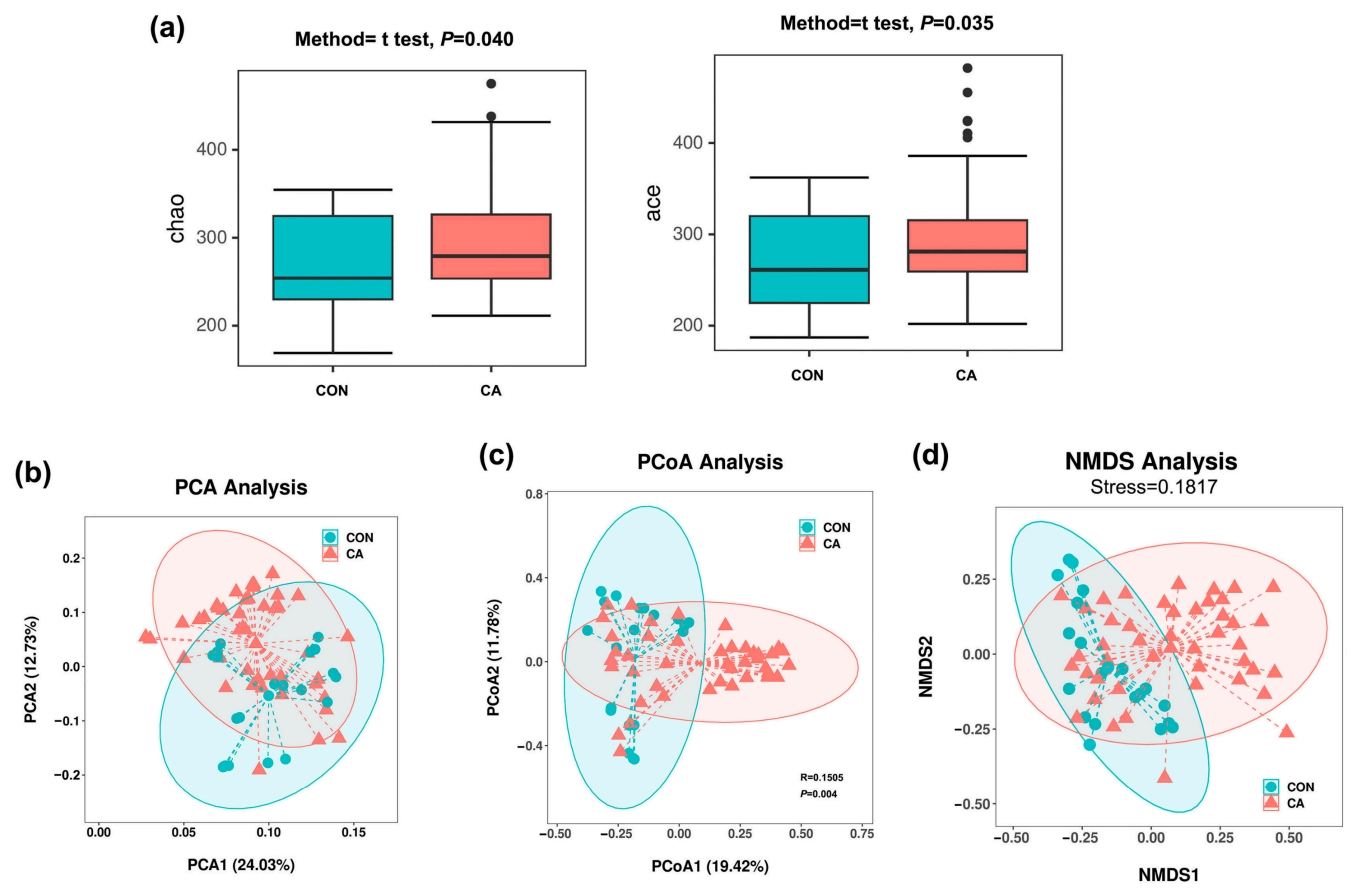
Condyloma acuminatum (CA) patients exhibit increased microbial richness and distinct gut microbial community structure compared with healthy controls (CON). (**a**) Chao1 and ACE richness estimators showing significantly higher community richness in the CA group (Chao1: *p* = 0.040; ACE: *p* = 0.035). (**b**) Principal component analysis (PCA) of operational taxonomic units (OTU)-level relative abundance between CON and CA groups (ANOSIM R = 0.134, *p* = 0.009). (**c**) Principal coordinate analysis (PCoA) based on weighted UniFrac distances between CON and CA groups (ANOSIM R = 0.151, *p* = 0.004). (**d**) Non-metric multidimensional scaling (NMDS) based on Bray–Curtis dissimilarities (stress = 0.182; ANOSIM R = 0.134, *p* = 0.01).

Microbial richness, as estimated using Chao1 and ACE indices, was significantly higher in the CA group compared with healthy controls (Figure 1a; Chao1: *p* = 0.040; ACE: *p* = 0.035). No significant differences were observed in Shannon, Simpson, or PD-whole tree indices between groups.

To assess overall microbial community differences, PCA was applied to OTU-level relative abundance data from the CON and CA groups (Figure 1b, ANOSIM R = 0.134, *p* = 0.009). PCoA utilizing weighted UniFrac distances disclosed a statistically slight segregation of microbial community structures between the CA and CON groups (Figure 1c; ANOSIM R = 0.151, *p* = 0.004). NMDS based on Bray–Curtis dissimilarities corroborated this structural divergence (Figure 1d; stress = 0.182, ANOSIM R = 0.134, *p* = 0.01).

### 3.2. CA Patients Display Altered Gut Microbiota Taxonomic Composition Compared with Healthy Controls

**Figure 2:**
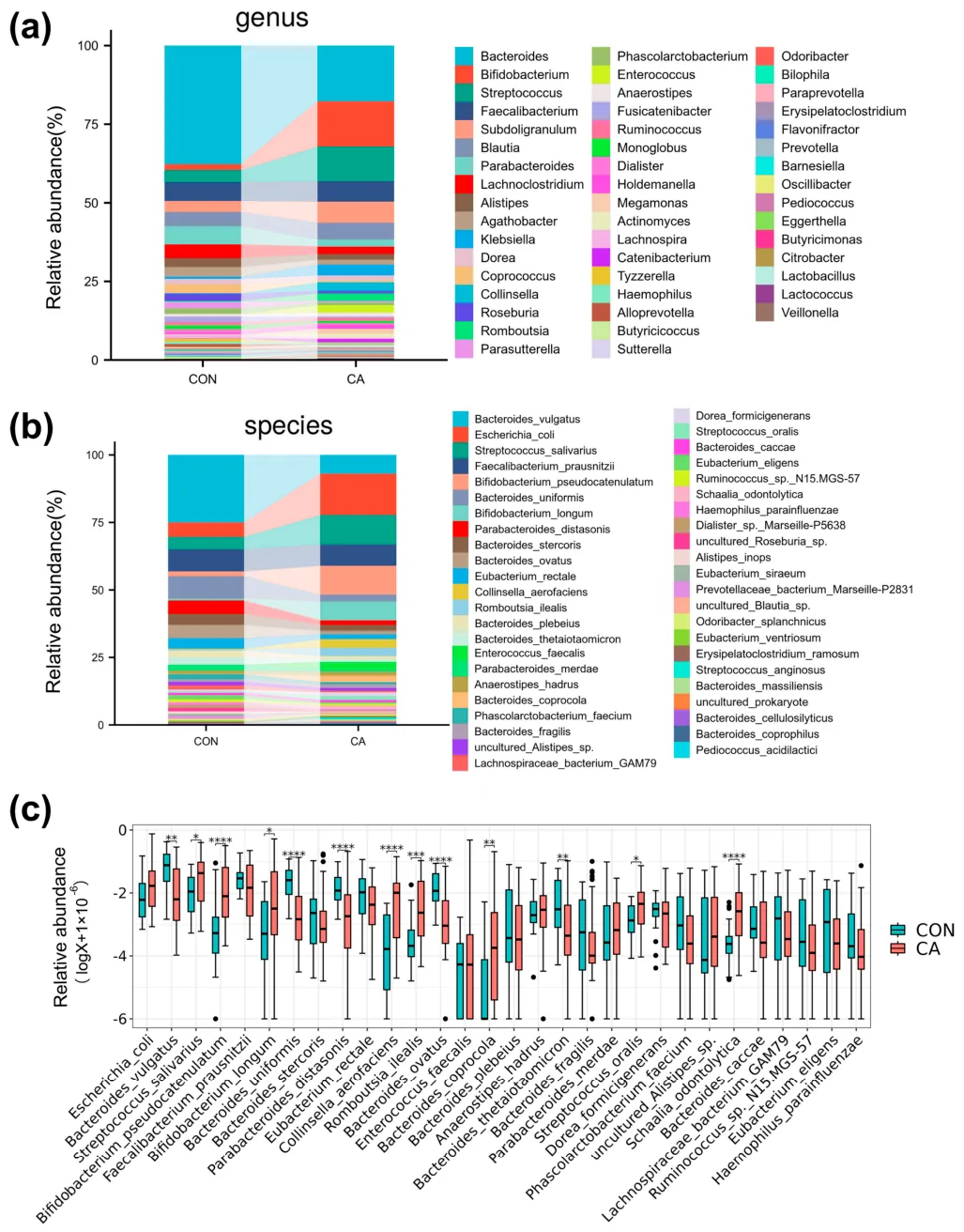
CA patients display altered gut microbiota taxonomic composition compared with healthy controls. (**a**) Relative abundance of gut microbial communities at the genus level in CON and CA groups, showing *Bacteroides* dominance in both groups but differential representation of *Parabacteroides*, *Bifidobacterium*, and *Faecalibacterium*. (**b**) Relative abundance at the species level, revealing reduced *Bacteroides* members (including *B. vulgatus*, *B. uniformis*, *P. distasonis*, *B. stercoris*, and *B. ovatus*) and elevated *S. salivarius*, *B. pseudocatenulatum*, and *B. longum* in the CA group. (**c**) Boxplots of the top 30 species with Wilcoxon rank-sum test results confirming significant abundance differences between groups. * *p* < 0.05; ** *p* < 0.01; *** *p* < 0.001; **** *p* < 0.0001.

At the genus level, notable compositional differences were observed between the CON and CA groups. The CON group was dominated by *Bacteroides*, *Parabacteroides*, *Faecalibacterium*, *Streptococcus*, and *Subdoligranulum*, whereas the CA group displayed a reshaped profile with *Bacteroides*, *Bifidobacterium*, *Streptococcus*, *Subdoligranulum*, and *Faecalibacterium* as the leading genera (Figure 2a). At the species level, several *Bacteroides* members—including *B. vulgatus*, *B. uniformis*, *P. distasonis*, *B. stercoris*, and *B. ovatus*—were reduced in the CA group relative to the CON group. In contrast, *S. salivarius*, *B. pseudocatenulatum*, *B. longum*, *C. aerofaciens*, and *R. ilealis* showed elevated abundance in CA patients (Figure 2b). Boxplot visualization of the top 30 species confirmed these distributional shifts. Statistical evaluation via the Wilcoxon rank-sum test verified significant abundance differences for multiple species between the CON and CA groups, reinforcing the genus- and species-level compositional alterations (Figure 2c).

### 3.3. Bacteroides stercoris Is Identified as an FDR-Significant LEfSe Biomarker for HPV Persistence, While Bacteroides vulgatus Shows Nominal Enrichment

**Figure 3:**
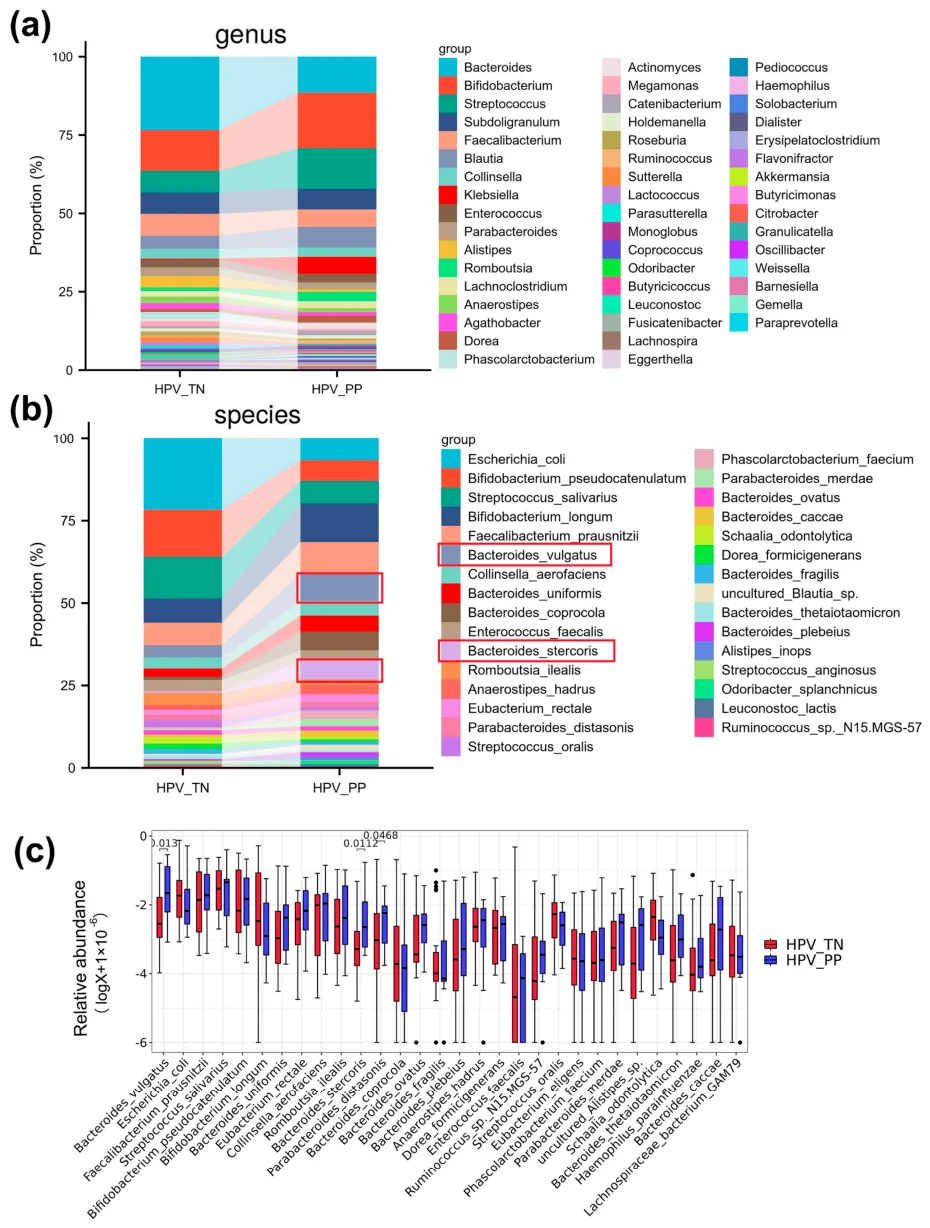
Gut microbial composition differs between HPV persistent-positve (HPV_PP) and HPV turn-negative (HPV_TN) groups. (**a**) Relative abundance at the genus level in HPV_PP and HPV_TN groups, showing shared dominant genera (*Bacteroides*, *Bifidobacterium*, *Streptococcus*, *Subdoligranulum*, *Faecalibacterium*). (**b**) Relative abundance at the species level, revealing higher abundances of *B. vulgatus* and *B. stercoris* in the HPV_PP group. (**c**) Boxplots of the top 30 species with the Wilcoxon rank-sum test showing nominally higher abundances of *B. vulgatus* (*p* = 0.014, *q* = 0.209), *B. stercoris* (*p* = 0.012, *q* = 0.209), and *P. distasonis* (*p* = 0.048, *q* = 0.512) in the HPV_PP group; none of the Wilcoxon-based differences survived false discovery rate (FDR) correction.

When comparing the HPV_TN and HPV_PP subgroups, both groups shared the same top five genera—*Bacteroides*, *Bifidobacterium*, *Streptococcus*, *Subdoligranulum*, and *Faecalibacterium* (Figure 3a)—but diverged notably at the species level. Specifically, *B. vulgatus*, *B. stercoris*, and *P. distasonis* displayed higher relative abundances in the HPV_PP group (Figure 3b). Boxplot comparisons across the top 30 species validated these observations. Wilcoxon rank-sum testing indicated nominally higher abundances of *B. vulgatus* (*p* = 0.014, *q* = 0.209), *B. stercoris* (*p* = 0.012, *q* = 0.209), and P. distasonis (*p* = 0.048, *q* = 0.512) in the HPV_PP group (Figure 3c). After Benjamini–Hochberg FDR correction, none of the Wilcoxon-based species differences reached statistical significance (all *q* > 0.05).

**Figure 4:**
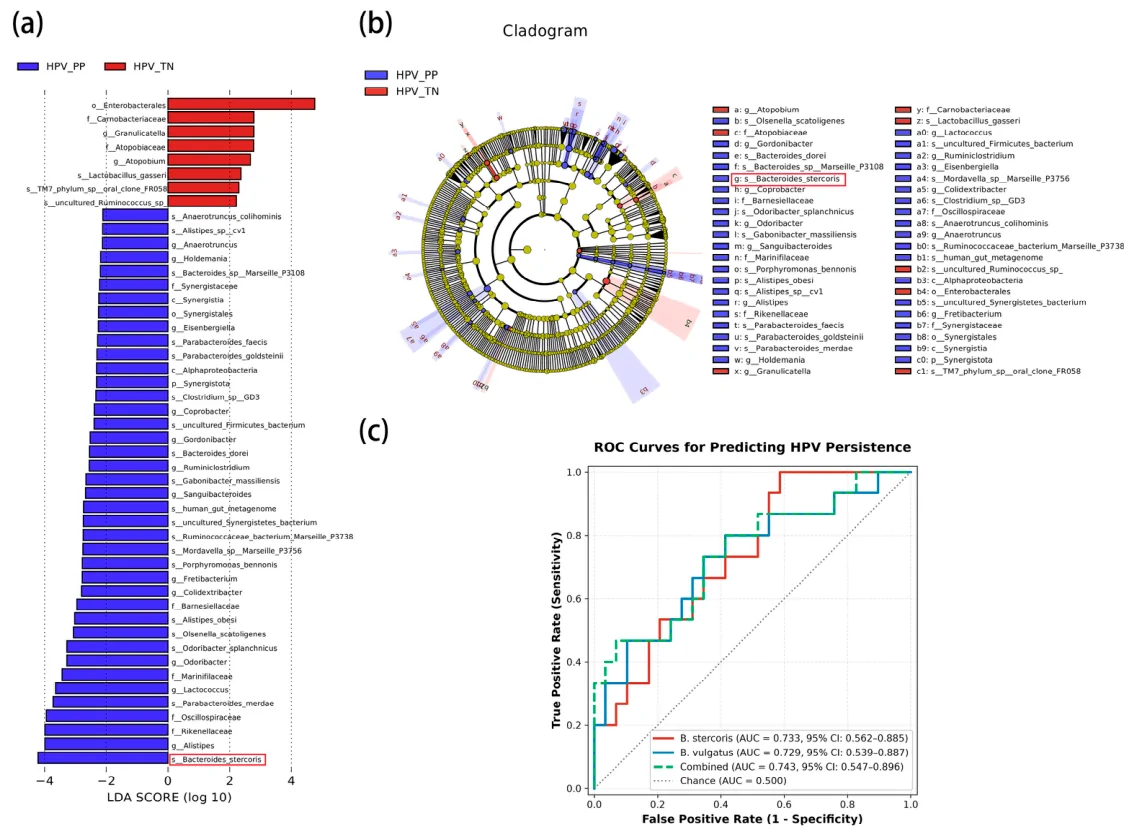
Linear discriminant analysis Effect Size (LEfSe) identifies *B. stercoris* as an FDR-significant biomarker and exploratory receiver operating characteristic (ROC) analysis assesses predictive value for HPV persistence. (**a**) Differentially abundant taxa between HPV_TN and HPV_PP groups selected via LEfSe (|LDA score| > 2 and *p* < 0.05), with Benjamini–Hochberg FDR correction applied. *B. stercoris* is the most robust species-level biomarker in HPV_PP (LDA = 4.21, *p* = 0.021, *q* = 0.034). (**b**) Cladogram showing phylogenetic distribution of the most discriminating taxa from phylum to species level (blue: HPV_PP-associated; red: HPV_TN-associated). (**c**) ROC curves showing exploratory predictive performance: individual area under the curves (AUCs) of 0.733 (*B. stercoris*, 95% CI: 0.562–0.885) and 0.729 (*B. vulgatus*, 95% CI: 0.539–0.887), with a combined two-species logistic regression model (*B. stercoris* + *B. vulgatus*) yielding AUC = 0.743 (95% CI: 0.547–0.896). These values are exploratory and require external validation.

Subsequently, LEfSe analysis was employed to uncover taxonomic biomarkers discriminating the HPV_TN and HPV_PP groups, with Benjamini–Hochberg FDR correction applied to the resulting *p*-values. The HPV_TN group displayed enrichment of *L. gasseri*, *TM7 phylum* sp., *oral clone FR058*, and *uncultured Ruminococcus* sp. The HPV_PP group, on the other hand, was characterized by higher levels of *B. stercoris*, *Parabacteroides merdae*, *O. splanchnicus*, *O. scatoligenes*, *A. obesi*, and additional taxa (Figure 4a, |LDA score| > 2 and *p* < 0.05). Notably, *B. stercoris* emerged as the most robust species-level biomarker in the HPV_PP group, with an LDA score of 4.21 and statistical significance that survived FDR correction (*p* = 0.021, *q* = 0.034). In contrast, *B. vulgatus* did not pass the LEfSe selection criteria (|LDA score| > 2). The cladogram provides a phylogenetic overview of the most discriminating taxa, delineating the lineages significantly associated with each group, blue for HPV_PP-associated taxa and red for HPV_TN-associated taxa, spanning from phylum to species level (Figure 4b).

To further explore whether specific species could serve as candidate predictors of persistent HPV infection, ROC curve analysis was undertaken. Individual AUC values were 0.733 for *B. stercoris* (95% CI: 0.562–0.885) and 0.729 for *B. vulgatus* (95% CI: 0.539–0.887) (Figure 4c). To determine whether a multi-species model might enhance discrimination, a combined logistic regression model integrating *B. stercoris* and *B. vulgatus* was constructed, yielding an AUC of 0.743 (95% CI: 0.547–0.896).

### 3.4. B. vulgatus and B. stercoris Are Positively Associated with HPV Persistence and Co-Occur with Other Bacteroides Species

**Figure 5:**
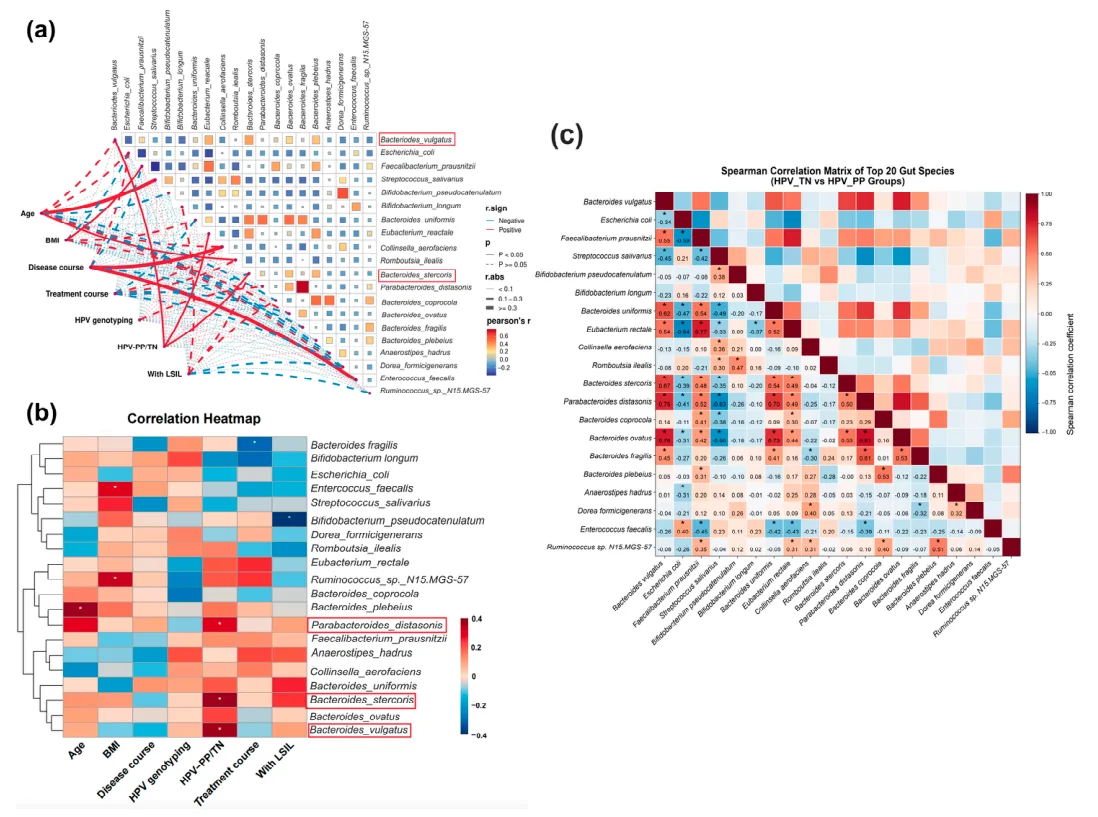
*B. vulgatus* and *B. stercoris* are positively associated with HPV persistence status and co-occur with other *Bacteroides* species. (**a**) Mantel’s test with Pearson rank correlation showing associations between the top 20 species and clinical characteristics, including age, body mass index (BMI), disease course, treatment duration, HPV genotype, status of low-grade squamous intraepithelial lesion (LSIL), HPV_PP status, with *F. prausnitzii* positively associated with BMI, C. aerofaciens and *E. faecalis* with disease duration, and *B. vulgatus*, *B. stercoris*, and *E. rectale* with HPV_PP status. Solid lines represent statistically significant correlations (*p* < 0.05), with red indicating positive and blue indicating negative associations. (**b**) Heatmap of Spearman’s rank correlation coefficients between the top 20 species and clinical features, confirming positive associations of *B. vulgatus* and *B. stercoris* with HPV_PP status (*, *p* < 0.05). (**c**) Co-occurrence network of the top 20 species showing a densely interconnected *Bacteroides* module (*B. vulgatus*, *B. stercoris*, *P. distasonis*, *B. ovatus*, *B. uniformis*; r = 0.50–0.81, *, *p* < 0.05).

Mantel’s test incorporating Pearson rank correlation coefficients was utilized to evaluate associations between the relative abundance of the top 20 species and clinical parameters. As depicted in Figure 5a, *F. prausnitzii* showed a positive association with BMI (*p* < 0.05), while *C. aerofaciens* and *E. faecalis* exhibited positive correlations with disease duration (*p* < 0.05). Among categorical variables, *B. vulgatus*, *B. stercoris*, and *E. rectale* were positively linked to HPV_PP status (persistent infection) (*p* < 0.05). The heatmap representation of Spearman’s rank correlation coefficients between the top 20 species and clinical features confirmed that *B. vulgatus* and *B. stercoris* maintained positive associations with HPV_PP status (Figure 5b, *p* < 0.05).

Inter-species Spearman correlation analysis among the top 20 species revealed that *B. vulgatus*, *B. stercoris*, *P. distasonis*, *B. ovatus*, and *B. uniformis* constituted a densely interconnected co-occurrence module, with pairwise correlation coefficients spanning 0.50 to 0.81 (Figure 5c, *p* < 0.05). In contrast, *S. salivarius* displayed significant inverse correlations with this *Bacteroides* cluster (r = −0.35 to −0.63, *p* < 0.05), and *B. longum* was negatively associated with *E. rectale* (r = −0.37, *p* < 0.05).

### 3.5. PICRUSt2 Predicts Differential Functional Pathway Enrichment Between HPV _PP and HPV_TN Groups

**Figure 6:**
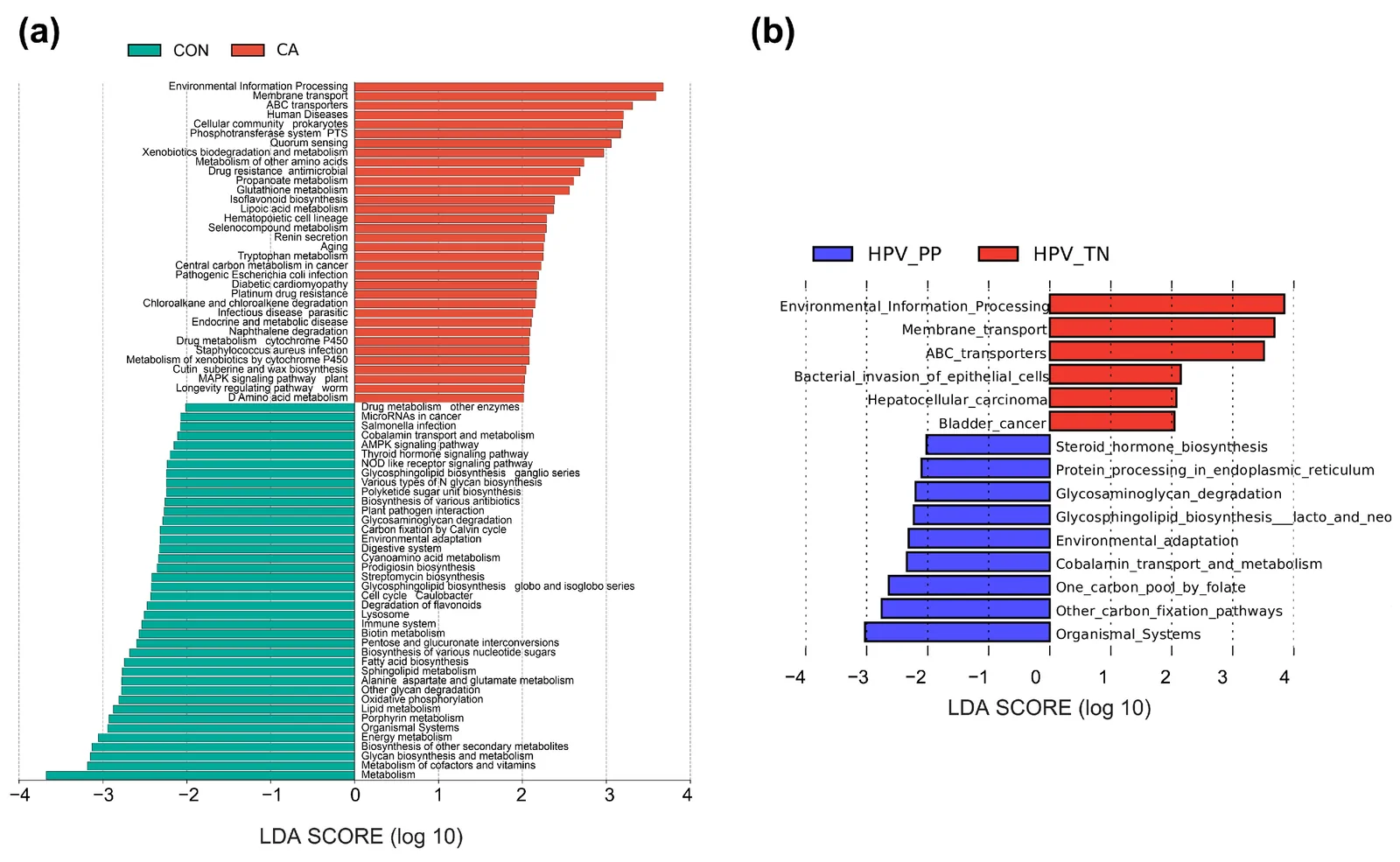
Phylogenetic Investigation of Communities by Reconstruction of Unobserved States (PICRUSt2) predicts differential Kyoto Encyclopedia of Genes and Genomes (KEGG) functional module enrichment between the CON and CA groups and between the HPV_PP and HPV_TN groups. (**a**) Significantly different KEGG modules between CA and healthy control groups, indicating predicted functional remodeling associated with CA. (**b**) Significantly different KEGG modules between HPV_TN and HPV_PP groups (*p* < 0.05, LDA score > 2, and *q* < 0.05), showing predicted enrichment of one-carbon pool by folate, cobalamin transport and metabolism, environmental adaptation, glycosphingolipid biosynthesis, and glycosaminoglycan degradation in HPV_PP and predicted reduction in ABC transporters, membrane transport, and environmental information processing.

PICRUSt2-based functional prediction identified KEGG module differences between the CA and CON groups (Figure 6a). Between the HPV_TN and HPV_PP groups, 15 KEGG categories differed significantly (*p* < 0.05, LDA score > 2, and *q* < 0.05; Figure 6b), with predicted enrichment of one-carbon pool by folate (*p* = 0.005, *q* = 0.033), cobalamin transport and metabolism (*p* = 0.047, *q* = 0.047), environmental adaptation, glycosphingolipid biosynthesis, and glycosaminoglycan degradation in the HPV_PP group and predicted depletion of ABC transporters, membrane transport, and environmental information processing.

## 4. Discussion

Although CA itself is generally benign, sustained HPV infection, especially with high-risk genotypes, constitutes a well-documented risk factor for anogenital cancer development [1,24]. The clinical management of CA continues to be hampered by frequent recurrence and an absence of dependable biomarkers for predicting whether patients will achieve viral clearance or develop persistence [2,3]. A growing body of evidence emphasizes the central role of host immunity in governing HPV outcomes, and the intestinal microbiome has attracted increasing attention as a crucial regulator of systemic immune competence [4,5,7].

In the current investigation, we conducted a comprehensive characterization of the gut microbiota in 44 female CA patients and 20 healthy controls through 16S rRNA gene sequencing, incorporating a 2-year follow-up to classify patients into HPV persistence (HPV_PP) and clearance (HPV_TN) subgroups. Our analyses uncovered significant shifts in microbial richness and composition between CA patients and controls and identified *B. stercoris* as a significant biomarker enriched in the HPV_PP group through LEfSe analysis after FDR correction. *B. vulgatus* showed nominally higher abundance in HPV_PP through Wilcoxon testing but did not pass LEfSe selection criteria. Both species demonstrated exploratory predictive value for persistent infection through ROC analysis. Spearman correlation analysis further demonstrated that these *Bacteroides* species co-occur with other *Bacteroides* species (r > 0.50). These findings suggest an association between intestinal microbial composition and HPV persistence; however, given the observational design with only baseline stool sampling, causality cannot be established, and the observed microbial profiles may reflect underlying differences in host immunity, lifestyle factors, or disease severity rather than directly driving viral persistence.

The enrichment of *B. stercoris* in the HPV_PP group was supported by both LEfSe analysis and Wilcoxon rank-sum testing, making it the most robust species-level association identified in this study. As a potential propionate producer via the succinate pathway [25], *B. stercoris*-derived propionate has been shown in other contexts to dampen Th1 polarization and IFN-γ secretion through HDAC inhibition and GPR43-mediated Treg expansion [8,26,27]—both of which are critical effectors for HPV clearance. Recent investigations have demonstrated that Bacteroides methylmalonyl-CoA mutase serves as a key enzyme in propionate generation, promoting intestinal goblet cell differentiation and mucosal barrier integrity [28]. Moreover, *Bacteroides*-derived outer membrane vesicles have been shown to modulate innate immune signaling cascades, including the cGAS-STING-type I interferon axis that governs antiviral defense [29,30]. Whether these mechanisms are operative in the context of HPV persistence in CA patients remains unknown and warrants mechanistic investigation.

*B. vulgatus*, which showed nominally higher abundance in the HPV_PP group through Wilcoxon testing but did not pass LEfSe selection criteria, has been reported in the literature to modulate host immunity through several pathways. It can produce butyric acid, which acts on the TGF-β1/MAPK signaling cascade implicated in immune suppression and tissue fibrosis [31]. Its lipopolysaccharide is recognized by the dendritic cell-specific intercellular adhesion molecule-3-grabbing non-integrin (DC-SIGN) receptor, a key lectin involved in immune homeostasis, suggesting a potential impact on antigen-presenting cell function and T-cell polarization [32]. Additionally, B. vulgatus may influence SCFA and bile acid metabolism, thereby affecting epithelial barrier integrity [33]. Compromise of the gut barrier could enhance intestinal permeability, enabling microbial products such as LPS to translocate into systemic circulation and drive low-grade inflammation [24], which might promote a shift from a Th1-dominant (antiviral) to a Th2-dominant (anti-inflammatory) response that is less effective at eliminating intracellular pathogens like HPV. The co-occurrence of *B. vulgatus* with *B. stercoris* and other *Bacteroides* species observed in our Spearman analysis is consistent with shared ecological niches; however, this co-occurrence pattern does not establish functional synergy, and the combined ROC model (AUC = 0.743) should be interpreted as exploratory given the limited sample size. It should be emphasized that the evidence for *B. vulgatus* is weaker than for *B. stercoris*, as its between-group difference did not survive FDR correction and it was not identified through LEfSe.

PICRUSt2 analysis predicted enrichment of folate biosynthesis and cobalamin transport pathways in the HPV_PP group. Folate and vitamin B12 serve as essential cofactors for DNA methylation and nucleotide synthesis—processes relevant to HPV replication and epigenetic reprogramming of infected keratinocytes [34,35]. The enrichment of *Bacteroides* species may synergistically reinforce immune suppression by promoting Treg induction and inhibiting cytotoxic T-cell responses [36]. These predicted differences provide a plausible mechanistic link between gut microbiota and persistent HPV infection. However, these are in silico predictions requiring metabolomic validation, and the proposed immune-modulatory and gut–vaginal axis mechanisms remain speculative.

In summary, our findings highlight *B. stercoris* as the most robust gut microbial biomarker associated with HPV persistence status in CA patients, supported by LEfSe analysis after FDR correction, nominal Wilcoxon significance, and exploratory ROC performance. *B. vulgatus* provides supplementary evidence, with nominally higher abundance in HPV_PP and comparable ROC performance, although it did not survive FDR correction or pass LEfSe selection. The combined two-species logistic regression model achieved an AUC of 0.743, suggesting modest predictive value. Complementing these taxonomic findings, PICRUSt2 functional prediction revealed 15 KEGG pathways predicted to be differentially enriched between the two groups, generating hypotheses regarding potential associations between gut microbial functional potential and HPV persistence. Together, the differential abundance of *B. stercoris* and *B. vulgatus*, their exploratory diagnostic performance, and the associated predicted functional pathway alterations suggest that specific gut microbial signatures may be linked to HPV persistence, offering candidate biomarkers for future prospective studies investigating whether probiotic or dietary interventions could influence viral clearance.

Several limitations should be noted. First, the modest sample size and single-center setting constrain statistical power and external validity; the particularly small HPV_PP and HPV_TN subgroups necessitate that LEfSe, ROC, and correlation-network analyses be interpreted as hypothesis-generating rather than confirmatory. A priori sample size estimation was not undertaken, and conventional post hoc power calculations are not readily adaptable to the high-dimensional, compositional structure of microbiome data; consequently, the study is susceptible to type II (false-negative) errors. Second, despite a 2-year longitudinal clinical follow-up, only baseline fecal samples were profiled, and the cross-sectional sampling design precludes definitive causal inferences regarding the temporal relationship between gut microbial composition and HPV persistence; the observed microbial profiles could represent predictors of persistence, reflect underlying differences in host immunity, be associated with lifestyle or behavioral factors, or correlate with disease severity or other unmeasured variables. Prospective longitudinal microbiome sampling would be needed to determine whether microbial changes precede or result from chronic infection. Third, while PICRUSt2 provided valuable functional predictions, these inferences are based on 16S rRNA data rather than direct metagenomic or metabolomic profiling, and predicted pathway differences do not constitute evidence of actual microbial metabolic activity. Fourth, species-level taxonomic assignments based on V3-V4 16S rRNA sequencing with 97% OTU clustering may not reliably discriminate closely related species, particularly within the genus *Bacteroides*; higher-resolution methods such as ASV-based analysis or shotgun metagenomics would be needed for confident species-level identification. Fifth, the ROC predictive model was developed and evaluated in the same cohort without an independent validation set or internal cross-validation, and the reported AUC may be optimistic; external validation in independent cohorts is essential before clinical utility can be assessed. Sixth, despite matching for key demographic variables, potential confounders including dietary patterns, recent antibiotic exposure, probiotic use, smoking, alcohol consumption, sexual behavior, previous treatments, and other medications or comorbidities were not systematically incorporated into multivariable models; the modest sample size precluded robust multivariable adjustment, and residual confounding cannot be excluded. Antibiotic exposure, in particular, represents a major potential confounder when attributing gut microbial signatures to HPV persistence [37]. Seventh, no vaginal microbiome analysis was performed, and the gut–vaginal axis remains a biologically plausible but untested hypothesis. Finally, although *B. stercoris* was identified as a significant LEfSe biomarker after FDR correction, providing the strongest statistical evidence among the species-level associations, the nominally significant Wilcoxon-based differences in *B. vulgatus* between HPV_TN and HPV_PP groups did not survive FDR correction, and these findings should therefore be regarded as exploratory trends requiring confirmation in larger cohorts. Given the large number of taxa and comparisons, other reported associations may also represent false positives. Future multi-center investigations integrating shotgun metagenomics, metabolomics, longitudinal sampling, multivariable adjustment, and mechanistic experiments are warranted to validate these findings and establish causal relationships.

## 5. Conclusions

In conclusion, this exploratory study provides evidence of associations between gut microbiota composition and predicted functional potential and HPV persistence in patients with CA. We identified *B. stercoris* as the most robust microbial signature, demonstrating significant enrichment in the HPV_PP group through LEfSe analysis after FDR correction, alongside nominal Wilcoxon significance and exploratory ROC performance. *B. vulgatus* showed nominally higher abundance in the HPV_PP group and comparable ROC performance, providing supplementary but less statistically robust evidence. These taxonomic findings were accompanied by predicted alterations in folate and cobalamin metabolic pathways that may be associated with HPV persistence status. These findings should be interpreted as observational associations rather than evidence of causation, and the identified taxa represent candidate biomarkers requiring independent external validation. The study generates hypotheses for future prospective and mechanistic investigations into whether gut microbial composition influences host immune responses relevant to viral clearance and whether microbiome-based interventions could have therapeutic potential. Larger multi-center cohorts with longitudinal microbiome sampling, multivariable confounder adjustment, shotgun metagenomics, and functional validation are needed to confirm and extend these findings.

## Data Availability

All raw sequencing reads have been deposited in the EMBL Sequence Read Archive under accession number PRJNA1033799.

## Supplementary Materials

The following supporting information can be downloaded at https://www.mdpi.com/article/10.3390/jcm15187265/s1, Table S1: Clinical characteristics of each patients.

## Abbreviations

The following abbreviations are used in this manuscript:

HPV	Human papillomavirus
CA	Condyloma acuminatum
CON	Control
FDR	False discovery rate
HPV_TN	HPV turn-negative
HPV_PP	HPV persistent-positive
SCFAs	Short-chain fatty acids
BMI	Body mass index
CI	Confidence intervals
PCA	Principal component analysis
PCoA	Principal coordinate analysis
OTU	Operational taxonomic units
LEfSe	LDA Effect Size
ROC	Receiver operating characteristic
AUC	Area under the curve
LDA	Linear discriminant analysis
KEGG	Kyoto Encyclopedia of Genes and Genomes
PICRUSt2	Phylogenetic Investigation of Communities by Reconstruction of Unobserved States
LSIL	Low-grade squamous intraepithelial lesion
